# Tongue lesions in psoriasis: a controlled study

**DOI:** 10.1186/1471-5945-4-16

**Published:** 2004-11-04

**Authors:** Maryam Daneshpazhooh, Homayoon Moslehi, Maryam Akhyani, Marjan Etesami

**Affiliations:** 1 Department of Dermatology, Tehran University of Medical Sciences, RAZI Hospital, Vahdate-Eslami Sq. 11966 Tehran, Iran

## Abstract

**Background:**

Our objective was to study tongue lesions and their significance in psoriatic patients.

**Methods:**

The oral mucosa was examined in 200 psoriatic patients presenting to Razi Hospital in Tehran, Iran, and 200 matched controls.

**Results:**

Fissured tongue (FT) and benign migratory glossitis (BMG) were the two most frequent findings. FT was seen more frequently in psoriatic patients (n = 66, 33%) than the control group (n = 19, 9.5%) [odds ratio (OR): 4.69; 95% confidence interval (CI): 2.61–8.52] (p-value < 0.0001). BMG, too, was significantly more frequent in psoriatic patients (28 cases, 14%) than the control group (12 cases, 6%) (OR: 2.55; 95% CI: 1.20–5.50) (p-value < 0.012). In 11 patients (5.5%), FT and BMG coexisted.

FT was more frequent in pustular psoriasis (7 cases, 53.8%) than erythemato-squamous types (56 cases, 30.4%). On the other hand, the frequency of BMG increased with the severity of psoriasis in plaque-type psoriasis assessed by psoriasis area and severity index (PASI) score.

**Conclusions:**

Nonspecific tongue lesions are frequently observed in psoriasis. Further studies are recommended to substantiate the clinical significance of these seemingly nonspecific findings in suspected psoriatic cases.

## Background

The occurrence of psoriatic lesions on oral mucous membranes was a subject of controversy [1,2]. Some investigators stated that they do not occur [3]; others, have claimed that they are uncommon. Still others say that they occur only in generalized pustular psoriasis (GPP) [4,5]. Nowadays, there is sufficient evidence that a subset of patients have oral lesions in association with skin disease [2].

Oppenheim, in 1903, was the first to substantiate oral psoriasis with biopsy [6]. Since then, various lesions have been described, including grey, yellowish, white or translucent plaques or annular forms, diffuse areas of erythema, geographic tongue and fissured tongue [7-16].

In all the cases reported in the literature, a positive biopsy showing a psoriasiform pattern has been the crucial component of the diagnosis [5,17-20]. Thus hyperkeratosis, parakeratosis, and an inflammatory infiltrate consisting of lymphocytes, polymorphonuclear leukocytes and histiocytes have been noted as well as Munro's microabscesses and spongiform pustules of Kogoj. In addition, many investigators believe that the presence of cutaneous lesions with a course parallel to that of oral lesions is necessary for establishing the diagnosis of oral psoriasis [2,5,20].

However, it is impossible to perform an oral biopsy in psoriatic cases in everyday clinical practice. On the other hand, some of the lesions seen more frequently in psoriatic patients are not specific histologically. In fact, similar changes are seen in otherwise healthy people (although with a lower frequency) leading to an underestimation of the value of these findings in psoriatic patients.

In order to substantiate further the relationship between these oral disorders and psoriasis, we compared 200 patients with psoriasis to a matched control group.

## Methods

Two hundred psoriatic patients (70 women and 130 men) attending the dermatology clinics of Razi Hospital, a major referral center in Tehran, from September 2000 till February 2001, were enrolled in this study using simple nonrandom (sequential) sampling. The diagnosis was made mainly on clinical data. The control group included 200 healthy subjects among the visitors of Surgery wards in a general hospital, matched one by one for age and sex. The skin and oral mucosa were examined in the two groups and, in addition to demographic and clinical data, PASI score [21] was recorded in plaque-type psoriasis. The data were analyzed by Epi-Info (version 6) software, and frequency, mean, standard deviation, OR and p-value were calculated.

## Results

The mean age of the patient group was 33.8+/- 18.2 years (4–79 years). The mean age of onset of disease was 26+/-17.7 years (0–74 years), 23 +/-18.8 years in women and 27.6 +/- 17.0 years in men. Age and sex were matched between patients and control subjects. Family history of psoriasis was positive in 34 patients. Different clinical types of psoriasis were as follows: Chronic plaque-type psoriasis (n = 140); generalized pustular psoriasis (n = 10); flexural psoriasis (n = 10); erythrodermic psoriasis (n = 9); localized pustular psoriasis (n = 3); guttate psoriasis (n = 9); palmoplantar psoriasis (n = 15); scalp (n = 95); nail alone (n = 3).

Oral findings were detected in 87 (43.5%) and 39 (19.5%) cases in the psoriatic and control groups, respectively. They are presented in table 1. FT was seen more frequently in psoriatic patients (66 patients, 33%) than the control group (19, 9.5%) (OR: 4.69; 95% CI: 2.61–8.52) (p-value < 0.0001). BMG, too, was significantly more frequent in psoriatic patients (28 cases, 14%) than the control group (12, 6%) (OR: 2.55; 95% CI: 1.20–5.50) (p-value < 0.012). BMG was seen in 18.2% of patients with FT, and 42.9% of patients with BMG suffered from FT. In other words, in 12 patients (6%) FT and BMG coexisted. In the control group, FT and BMG coexisted in 2 cases (1%).

**Table 1:** The frequency of oral findings in psoriasis patients and control group

	Psoriasis	Control
Fissured tongue	66	19
Benign migratory glossitis	28	12
Diffuse oral and tongue erythema	11	3
Hairy tongue	2	4
Rhomboid glossitis	2	3
Depapillated tongue	2	3

One hundred eighty-four patients (92%) suffered from erythemato-squamous lesions and 13 cases (6.5%) from pustular lesions. The frequency of FT in the erythemato-squamous and pustular groups was 30.4% (56 cases) and 53.8% (7 cases), respectively. On the other hand, the frequency of BMG in the erythemato-squamous and pustular groups was 14.1% (26 cases) and 15.4% (2 cases), respectively.

The severity of chronic plaque-type psoriasis cases assessed by PASI score was as follows: mild, 53 cases (37.9%); moderate, 60 cases (42.9%); and severe 27 cases (19.3%). The corresponding frequency of FT and BMG in the three severity groups is presented in table 2. The frequency of BMG increased with the severity of skin lesions (p-value < 0.001).

**Table 2:** Frequency of fissured tongue and benign migratory glossitis according to severity in plaque-type psoriasis

	Fissured tongue	Benign migratory glossitis
Mild	14 (26.4%)	3 (5.7%)
Moderate	23 (38.3%)	10 (16.7%)
Severe	7 (25%)	9 (32.1%)

## Discussion

In general oral lesions in psoriasis can be divided into two major categories. The first one includes authentic psoriatic lesions proved by biopsy and with a parallel clinical course with skin lesions. It's not known whether these lesions are truly rare, or they remain undetected, as mucosal biopsy is seldom done in known psoriatic cases. The second group comprises the majority of oral findings in psoriasis and includes nonspecific lesions such as FT and psoriasiform lesions such as BMG [22]. These lesions are underestimated in the literature, but deserve more attention due to their high frequency.

We will discuss the main oral findings observed in our study as well as those reported in the literature.

Fissured tongue, also termed lingua fissurata, lingua plicata, scrotal tongue, and grooved tongue is recognized clinically by an antero-posteriorly oriented fissure, often with branch fissures extending laterally. It's believed by most authors to be an inherited trait. The frequency of FT increases with age and has been associated with Down's syndrome and the Melkerson-Rosenthal syndrome [5,20].

According to our study, FT was the most common oral finding in the psoriasis group: Nearly one-third of patients suffered from FT. It was significantly more frequent in psoriasis patients than the control group (9.5%) (p-value < 0.0001). The previously reported figures of the frequency of FT in the general population vary markedly in the literature depending on the study design and the target study. Axell reported a figure of 6.5% [10] and Morris found FT in 20.3% of its target study [17]. Aboyans et al reported a frequency of 2.56% in Iran [23]. On the other hand, FT was reported in 6–16.7% of psoriatic patients in different studies [3,10,14,15,20].

BMG or geographic tongue presents clinically as one or more erythematous patches with a raised white or yellow serpiginous border. Lesions may migrate across the tongue by healing on one edge while extending on another. BMG has no known cause, but it has been associated with atopic conditions, diabetes mellitus, reactive bronchitis, anemia, stress [20], hormonal disturbances, Down's syndrome and lithium therapy [24]. Lesions identical to BMG have been described in patients with Reiter's syndrome and psoriasis. The association of both psoriasis and BMG with HLA-CW6 provides further evidence that the two disorders are related [25].

In our study, BMG was significantly more frequent in psoriatic patients (14%) than the control group (6%) (p-value < 0.012). According to the literature, the estimated frequency of BMG in the general population is from 1–5% [1,10,20,26] and varies from 1–10.3% in psoriatic patients [3,10,13-15,20,26]. Only Hietanen found a figure of 1% in psoriasis [10].

FT was more frequent in patients with pustular lesions compared with the erythemato-squamous types. Contrary to previous studies, this finding was not seen for BMG, a disease generally considered accompanied with GPP [27,28]. This may be due to the low frequency of GPP in our study group. On the other hand, the frequency of BMG increased with the severity of psoriasis in plaque-type disease, a finding not seen in Morris's study [20], perhaps due to different definition for the severity of the disease. According to our study, the frequency of FT didn't increase by increasing severity of psoriasis.

SAM was first described by Cooke in 1955 as an idiopathic inflammatory condition of the nonlingual oral mucosa [10,15]. It is also denoted using different terms: Geographic stomatitis, ectopic geographic tongue, erythema circinate migrans, and migratory stomatitis. These lesions are similar in appearance to BMG, but occur on the oral mucosal surfaces as well as the dorsum of the tongue [1,29,30].

As seen in Van der Wal's study, [14] we didn't find SAM in psoriatic patients. The reported frequency of this oral finding in psoriatic patients in the literature is between 0–19% [15,20]. Furthermore, this lesion seems to be very rare in the general population, too: Bouquot found no patients with SAM in 231616 white American dental patients [20].

Diffuse oral and tongue erythema was another positive finding in the psoriasis group with a frequency of 5.5%. This lesion, too, was reported previously in the literature, although with a lower frequency (1%) [10].

An association between FT and BMG is well established in the literature [31,32]. In our study, BMG was seen in 18.2% of patients with FT (results consistent with Pindorf's) [14,16].

## Conclusions

Overall, although oral lesions might not be considered authentic oral psoriasis unless proven histologically and with a parallel clinical course, nonspecific tongue lesions are significantly more frequent in psoriatic cases. Further studies are recommended to evaluate the clinical significance of these seemingly nonspecific lesions in a suspected psoriatic case. Furthermore, more thorough studies are recommended regarding the relationship of oral psoriasis and disease severity in plaque-type psoriasis.

## Competing interests

The author(s) declare that they have no competing interests.

## Authors' contributions

All authors contributed equally in the study design, literature search, data analysis and manuscript preparation. All authors read and approved the final manuscript.

## Pre-publication history

The pre-publication history for this paper can be accessed here:


